# Clustering Categorical Data Using Community Detection Techniques

**DOI:** 10.1155/2017/8986360

**Published:** 2017-12-21

**Authors:** Huu Hiep Nguyen

**Affiliations:** Institute of Research and Development, Duy Tan University, P809 7/25 Quang Trung, Danang 550000, Vietnam

## Abstract

With the advent of the *k*-modes algorithm, the toolbox for clustering categorical data has an efficient tool that scales linearly in the number of data items. However, random initialization of cluster centers in *k*-modes makes it hard to reach a good clustering without resorting to many trials. Recently proposed methods for better initialization are deterministic and reduce the clustering cost considerably. A variety of initialization methods differ in how the heuristics chooses the set of initial centers. In this paper, we address the clustering problem for categorical data from the perspective of community detection. Instead of initializing *k* modes and running several iterations, our scheme, CD-Clustering, builds an unweighted graph and detects highly cohesive groups of nodes using a fast community detection technique. The top-*k* detected communities by size will define the *k* modes. Evaluation on ten real categorical datasets shows that our method outperforms the existing initialization methods for *k*-modes in terms of accuracy, precision, and recall in most of the cases.

## 1. Introduction

Clustering task is a form of unsupervised learning that aims at finding underlying structures in unlabeled data. Objects are partitioned into homogeneous groups or clusters so that intracluster items have high similarity but are very dissimilar to objects in other clusters. A lot of clustering methods have been proposed and developed over decades (for a recent survey, see [1]).* Hierarchical* clustering and* partitional* clustering are two main types of clustering algorithms. While hierarchical clustering produces a hierarchy of partitions (i.e., a dendrogram) over the dataset by applying agglomerative or divisive strategies, partitional clustering usually assumes a fixed number of clusters and tries to maximize the homogeneity within the clusters.

For numerical data, the *k-means* algorithm is a well-known and widely used method in practice due to its simplicity and efficiency. *K*-means finds a set of *k* cluster centers for a dataset such that the sum of squared distances of each point to its nearest cluster center is minimized. Lloyd's algorithm [2] begins with *k* arbitrary centers, typically chosen uniformly at random from the data points. Each point is then assigned to the nearest center, and each center is recomputed as the center of mass of all points assigned to it. These two steps are repeated until the process stabilizes. To remove the numeric-only limitation of the *k*-means algorithm, Huang [3] developed the *k*-modes algorithm which extends the *k*-means algorithm by using (1) a simple matching dissimilarity measure for categorical attributes (2) modes in place of means for clustering and (3) a frequency-related strategy to update modes to minimize the clustering cost. The algorithm is shown to achieve convergence with linear time complexity with respect to the number of data items.

However, the *k*-modes algorithm is also very sensitive to the choice of initial cluster centers and an improper choice may result in highly undesirable cluster structures. The same phenomenon happened in *k*-means which led to the better seeding solutions such as* k-means++* [4] and its derivatives *k*-means‖ [5] and *k*-*MC*^2^ [6]. To better initialize cluster centers in *k*-modes, a lot of methods have been developed [7–10]. The common point of [7, 8, 10] is to use the density of each data point together with the distance to determine sequentially the *k* initial cluster centers. Khan and Ahmad [9] proposed to use multiple clustering of the data based on attribute values in different attributes.

In this paper, we develop a new clustering method for categorical data based on community detection techniques [11]. Considering each data point as a node, we build a simple graph *G* in which an edge connects any two nodes if the Hamming distance between them is less than a threshold. The threshold is simply estimated via the number of data points *N*, the number of clusters *K*, and the pairwise Hamming distance distribution. Given the graph *G*, we run the Louvain algorithm [12] to detect nonoverlapping cohesive communities within *G*. The top-*K* communities by size will be retained as the core clusters, each of which is represented by a mode. The remaining data points (if any) are assigned to the nearest mode. Note that our algorithm is not an initialization technique as [3, 7–10] because it produces clusters directly.

Compared to prior work, our scheme highlights the following features:

- We propose a novel clustering method called CD-Clustering for categorical data using community detection techniques. Our scheme uses a simple heuristic to determine the distance threshold for graph construction. It is also deterministic as opposed to the traditional *k*-modes with random initialization of cluster centers.
- We evaluate our scheme on ten real categorical datasets and compare it against random initialization and two other initialization methods. The results show that our technique performs better than the competitors in terms of accuracy for most of the cases.

The remainder of the paper is organized as follows.  Section 2 briefly reviews related work in *k*-modes clustering and community detection.  Section 3 discusses several key concepts used in this paper via some illustrative examples. In Section 4, we describe a simple estimation of the distance threshold and our main algorithm. The evaluation and comparison are shown in Section 5. Finally, Section 6 concludes the paper with pointers to future work.

## 2. Related Work

### 2.1. *K*-Modes and Initialization Techniques

As in *k*-means, the random initialization method has been widely used in *k*-modes clustering for its simplicity. However, the random method does not guarantee a unique clustering result, and very poor clustering results may occur. To obtain desirable clustering results with low distortion, the *k*-modes algorithm must be executed many times.

In [3], Huang proposed two simple initialization methods for *k*-modes, in which the first method selects the first *k* objects from the dataset as initial cluster centers, and the second method assigns the most frequent categories equally to the *k* initial cluster centers. However, the first method works only if the first *k* objects come from *k* disjoint clusters while the second method lacks a uniform criterion for selecting initial clusters.

Wu et al. [10] proposed a density based initialization method for *k*-modes. Cao et al. [8] presented a method to select initial cluster centers by considering the distance between objects and the density of each object. Bai et al. [7] proposed an initialization method that is similar to [8] but tries to avoid selecting the boundary objects among clusters as the first cluster center. However, the evaluation of results in [7] has some problems: for several datasets, the accuracy, precision and recall values are computed incorrectly as reported by Khan and Ahmad [9]. In [9], Khan and Ahmad presented an initialization algorithm for *k*-modes by performing multiple clustering of data based on the attribute values present in different attributes.

A long with the heuristics for cluster initialization discussed above, there are many ideas on improving the dissimilarity scores for the standard *k*-modes algorithm [13–16]. Ng et al. [15] gave a rigorous proof that the object cluster membership assignment method and the mode updating formula under the dissimilarity measure proposed in [14] indeed minimize the objective function. Cao et al. [13] proposed a new dissimilarity measure to take into account of the distribution of attribute values on the whole universe. In [16], Zhou et al. took a step further by defining the Global-Relationship dissimilarity (GRD) measure.

### 2.2. Community Detection in Graphs

There is a vast literature on community detection in graphs. For a recent comprehensive survey, we refer to [11]. In this section, we discuss several classes of techniques.

Newman and Girvan [17] propose* modularity* as a quality of network clustering. It is based on the idea that a random graph is not expected to have a modular structure, so the possible existence of clusters is revealed by the comparison between the actual density of edges in a subgraph and the density one would expect to have in the subgraph if the nodes of the graph were connected randomly (the null model).

Many methods for optimizing the modularity have been proposed over the last ten years, such as agglomerative greedy [18], simulated annealing [19], spectral optimization [20], and Louvain method [12], just to name a few. Other methods include random walks [21], statistical mechanics [22], label propagation [23], and InfoMap [24]. The recent multilevel approach, also called* Louvain method*, by Blondel et al. [12] is among top performance schemes. It scales very well to graphs with hundreds of millions of nodes/edges.

## 3. Preliminaries

In this section, we review several key concepts in the *k*-modes algorithm and community detection techniques. We also discuss how the clustering problem of categorical data can be solved from the perspective of community detection.

Notations summarizes the notation used in this paper.

### 3.1. Clustering Categorical Data

Let *X* = {*X*_1_, *X*_2_,…, *X*_*N*_} be a categorical dataset with *N* data points *X*_*i*_. Each data point *X*_*i*_ has *M* categorical attributes from the set *A* = {*A*_1_, *A*_2_,…, *A*_*M*_}. In other words, the dataset *X* can be represented by a table with *N* rows and *M* columns in which *X*_*ij*_ ∈ *A*_*j*_ indicates the *j*th attribute of the data point *X*_*i*_.

The *k*-modes clustering algorithm [3] is an extension of the *k*-means algorithm for clustering categorical data by using a simple dissimilarity measure. It also adopts a frequency-related strategy to update modes in the clustering to minimize the clustering costs. The simplest matching dissimilarity measure between two data points *x* and *y* is defined by Hamming distance:

(1) $$\begin{array}{c} Dis\left(\;x,y\;\right)\equiv d_{H}\left(\;x,y\;\right)=\overset{M}{\underset{j=1}{\sum }}\left(\;1-\delta \left(x_{j},y_{j}\right)\;\right), \end{array}$$

where *x*_*j*_ denotes the *j*th attribute of *x* and *δ*(*x*_*j*_, *y*_*j*_) = 1 if *x*_*j*_ = *y*_*j*_ or *δ*(*x*_*j*_, *y*_*j*_) = 0 otherwise. Obviously, the Hamming distance between any two data points lies in the set {0,1,…, *M*}.

Given a set of data points *Y* = {*Y*_1_, *X*_2_,…, *Y*_*n*_}, a* mode* of *Y* is an object *Z* = [*z*_1_, *z*_2_,…, *z*_*M*_] where *z*_*j*_ ∈ *A*_*j*_ that minimizes the sum ∑_*i*=1_^*n*^*d*_*H*_(*Y*_*i*_, *Z*). In other words, *z*_*j*_  (1 ≤ *j* ≤ *M*) is the most frequent value in *Y* with respect to the *j*th attribute [3]. Note that *Z* is not necessarily an object of *Y*. When a mode is not an object of a set, it can be assumed as a virtual object.

The original *k*-modes algorithm [3] tries to minimize the following cost function:

(2) $$\begin{array}{c} P\left(\;W,Z\;\right)=\overset{K}{\underset{k=1}{\sum }}\;\overset{N}{\underset{i=1}{\sum }}w_{i,k}d_{H}\left(\;X_{i},Z_{k}\;\right), \end{array}$$

where *w*_*i*,*k*_ ∈ {0,1} and ∑_*k*=1_^*K*^*w*_*i*,*k*_ = 1  ∀ *i* = 1 ⋯ *N*. The *k*-modes algorithm [3] runs the following steps:

1. Select *K* initial modes, one for each cluster.
2. Allocate an object to the cluster whose mode is the nearest to it. Update the mode of the cluster after each allocation using the most frequent attribute values.
3. After all objects have been allocated to clusters, retest the dissimilarity of objects against the current modes. If an object is found such that its nearest mode belongs to another cluster rather than its current one, reallocate the object to that cluster, and update the modes of both clusters.
4. Repeat (3) until no object has changed clusters after a full cycle test of the whole dataset.

### 3.2. Community Detection via Modularity Optimization

Given a simple graph *G* with *n*_*c*_ disjoint communities, the modularity *Q* is defined as

(3) $$\begin{array}{c} Q=\overset{n_{c}}{\underset{c=1}{\sum }}\left[\;\frac{l_{c}}{m}-{\left(\frac{d_{c}}{2m}\right)}^{2}\;\right]. \end{array}$$

where *n*_*c*_ is the number of clusters, *l*_*c*_ is the total number of edges joining nodes in community *c*, and *d*_*c*_ is the sum of the degrees of the nodes of *c*. Modularity is a scalar value in the range (−1,1) with larger values implying better clustering.

**Example 1 .**

**Figure 1:**
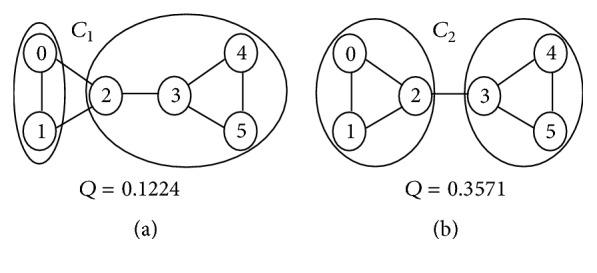
Modularity of two different clustering.

Using Figure 1, we illustrate how to compute the modularity of a graph *G* with respect to a clustering *C*. The graph has six nodes and seven edges (*m* = 7). In Figure 1(a), the clustering is *C*_1_ = {{0,1}, {2,3, 4,5}}, so *n*_*c*_ = 2 (two clusters). For the first cluster, *l*_1_ = 1, *d*_1_ = deg⁡(0) + deg⁡(1) = 4. For the second cluster, *l*_2_ = 4, *d*_2_ = deg⁡(2) + deg⁡(3) + deg⁡(4) + deg⁡(5) = 10. Hence, following formula (3), the modularity is *Q* = 1/7 − (4/(2×7))^2^ + 4/7 − (10/(2×7))^2^ = 0.1224.

Similarly, for the clustering on Figure 1(b)*C*_2_ = {{0,1, 2}, {3,4, 5}}, the modularity is *Q* = 3/7 − (7/(2×7))^2^ + 3/7 − (7/(2×7))^2^ = 0.3571. Clearly, the modularity of *C*_2_ is higher than that of *C*_1_. This fact is also confirmed by looking at the two types of clustering in which *C*_2_ partitions the nodes into more homogeneous groups.

Since its introduction in 2008,* Louvain method* [12] becomes one of the most cited methods for the community detection task. It optimizes the modularity by a bottom-up folding process. The algorithm is divided into passes, each of which is composed of two phases that are repeated iteratively. Initially, each node is assigned to a different community. So, there will be as many communities as there are nodes in the first phase. Then, for each node *i*, the method considers the gain of modularity if we move *i* from its community to the community of a neighbor *j* (a* local change*). The node *i* is then placed in the community for which this gain is maximum and positive (if any); otherwise it stays in its original community. This process is applied repeatedly and sequentially for all nodes until no further improvement can be achieved and the first pass is then complete.

**Example 2 .**

**Figure 2:**
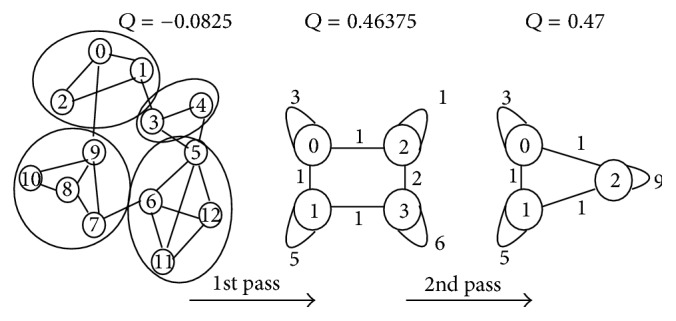
Louvain method for modularity optimization.

We demonstrate Louvain method in Figure 2 with a graph of 13 nodes and 20 edges. If each node forms its own singleton community, the modularity *Q* will be −0.0825. In the first pass of Louvain method, each node moves to the best community selected from its neighbors' communities. We get the partition [{0,1, 2}, {3,4}, {5,6, 11,12}, {7,8, 9,10}] with modularity 0.46375. The second phase of first pass builds a weighted graph corresponding to the partition by aggregating communities. The second pass repeats the folding process on this weighted graph to reach the final partition [{0,1, 2}, {3,4, 5,6, 11,12}, {7,8, 9,10}] with modularity 0.47.

This greedy agglomerative algorithm has several advantages as stated in [12]. First, its steps are intuitive and easy to implement, and the outcome is unsupervised. Second, the algorithm is extremely fast, that is, computer simulations on large modular networks suggest that its complexity is linear on typical and sparse data. This is due to the fact that the possible gains in modularity are easy to compute and the number of communities decreases drastically after just a few passes so that most of the running time is concentrated on the first iterations. Third, the multilevel nature of the method produces a hierarchical structure of communities which allows multiresolution analysis, that is, the user can zoom in the graph to observe its structure with the desired resolution.

Note that in Louvain method, the move of nodes to gain better modularity is restricted to neighbor (connected) communities. Therefore, detected communities belong to one and only one connected component. In other words, a community never spans different connected components of a graph.

## 4. Algorithm

### 4.1. Estimation of Hamming Distance Threshold

To build the graph *G* for the dataset *X*, we need to estimate the distance threshold *R* so that any two data points *x* and *y* are connected if the Hamming distance *d*_*H*_(*x*, *y*) ≤ *R*. As mentioned in Section 3.1, the Hamming distance *d*_*H*_(*x*, *y*) lies in the set {0,…, *M*}. At one extreme *R* = 0, the graph *G* has least edges which exist between duplicate data points only. At the other extreme *R* = *M*, we get a complete graph *G*: any two nodes are connected. Obviously, some values of the distance threshold *R* will make *G* look more* modular* than the others; that is, its nodes are well clustered in communities and therefore easier to detect.

In this paper, we propose a simple heuristic to estimate *R* based on the distribution of Hamming distances between data points in *X* given the number of clusters *K*. With *N* data points, there are *N*(*N* − 1)/2 pairwise distances. Trivially assuming that *K* clusters are of equal size, each cluster will have *N*/*K* points and the number of intracluster distances in each cluster is (*N*/*K*(*N*/*K* − 1))/2. In total, there are *K*((*N*/*K*(*N*/*K* − 1))/2) = *N*(*N* − *K*)/2*K* intracluster distances. In practice *K* ≪ *N*, so the ratio of intracluster distances over the number of pairwise distances is

(4) $$\begin{array}{c} \frac{N\left(N-K\right)/2K}{N\left(N-1\right)/2}\approx \frac{1}{K}. \end{array}$$

**Figure 3:**
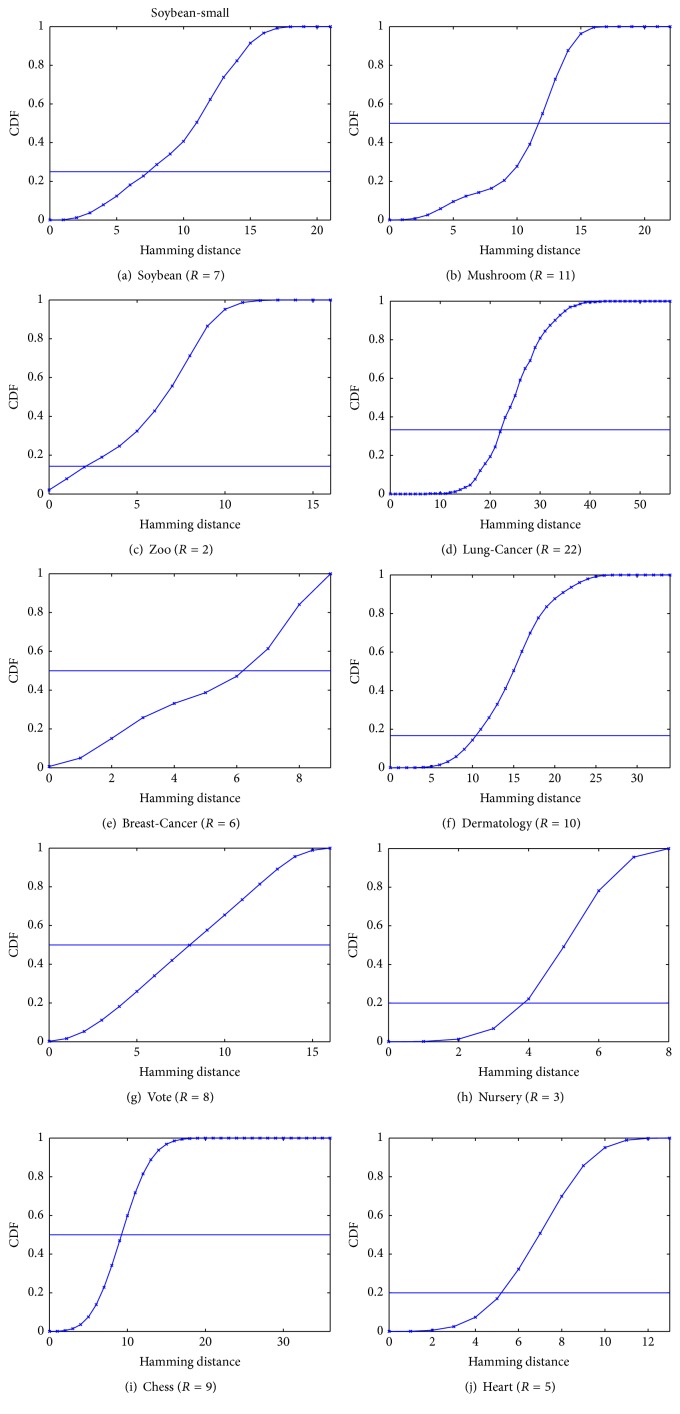
Estimation of the distance threshold *R*. The horizontal line in each figure represents 1/*K*.

In other words, given the cumulative distribution function (CDF) of pairwise distances, we can estimate *R* at the point that CDF(*R*) ≤ 1/*K* and CDF(*R* + 1) > 1/*K*.  Figure 3 illustrates this idea for the ten datasets used in our experiments.

We also observe that the* expected* Hamming distance between two random data points is large when the attribute values are assumed to be uniformly distributed. Specifically, given the set of attributes *A* = {*A*_1_, *A*_2_,…, *A*_*M*_}, the expected Hamming distance between two random data points *x* and *y* is

(5) EdHx,y=E∑j=1M1−δxj,yj=M−∑j=1MEδxj,yj=M−∑j=1M1Aj≥M−M2=M2,

where |*A*_*j*_| ≥ 2 is the cardinality of the *j*th nonsingleton attribute. The larger |*A*_*j*_|, the larger the expected Hamming distance.

### 4.2. Clustering Algorithm

Now we describe our community detection-based clustering scheme (named CD-Clustering) which is outlined in Algorithm 1. The scheme consists of two phases. In the first phase, we compute all pairwise Hamming distances and the CDF of distance distribution (Lines (1)-(2)). Then, we estimate the distance threshold *R* using a simple assumption in Section 4.1 (Lines (4)–(6)). In the second phase, we build the graph *G* in which each node represents a data point. Two nodes are connected by an edge if their Hamming distance is not larger than *R* (Lines (8)–(11)). In Line (12), we run the Louvain method [12] on *G* to detect highly cohesive groups of nodes. The top-*K*  {*C*_*k*_} detected communities by size will be retained (Line (13)). Then, we determine the mode *Z*_*k*_ for data points in each community *C*_*k*_ (Lines (14)-(15)). The remaining data points (i.e., data points that do not belong to any of the top-*K* communities) are assigned to the nearest mode (Lines (16)-(17)). As we show later in Section 5, except the dataset* Mushroom*, the number of remaining data points is very small.

The complexity of CD-Clustering is dominated by the computation of all pairwise Hamming distances and the Louvain method. All pairwise Hamming distances are computed in *O*(*N*^2^*M*). The Louvain method runs empirically in the time linear to the number of edges [12]. Again, using the simple assumption that all *K* clusters are of equal size, the number of intracluster distances is approximated as *N*(*N* − *K*)/2*K*. So the number of edges in *G* is also *N*(*N* − *K*)/2*K*, making the runtime of Louvain method is *O*(*N*^2^/*K*). In total, the complexity of CD-Clustering is *O*(*N*^2^*M* + *N*^2^/*K*). The quadratic complexity is a main drawback of our CD-Clustering scheme which restricts its application to datasets of 50,000 data points or less. A similar scalability limitation appears in [25] in which the authors need a similarity matrix of size *N*^2^. An approximation of Hamming distance distribution is possible by considering, for example, the distances from any point *x* to $O(\sqrt{N})$ (instead of all *N* − 1) other points. The complexity in this approximation scheme will be reduced to *O*(*N*^1.5^*M*). We leave this idea for future work.  Table 1 compares the time complexity of our CD-Clustering scheme with the two initialization methods [8, 9].

## 5. Evaluation

In this section, we evaluate the performance of the proposed scheme. The real-world datasets and evaluation metrics are described in Sections 5.1 and 5.2. We show the performance of our method in Section 5.3. The clustering algorithm is implemented in C++ and run on a desktop PC with* Intel*® Core i7-6700@ 3.4 Ghz, 16 GB memory. For the sake of reproducibility, we provide our source code with the data (https://gitlab.com/hiepnh.duytan/Research/tree/master/k-modes-community).

### 5.1. Datasets

We pick ten purely categorical datasets from the UCI Machine Learning Repository [26] with a short description for each dataset as follows. Compared to the datasets used in [9], we add three new datasets: Nursery, Chess, and Heart. Note that we consider missing attribute values “?” as a new attribute value.

*Soybean Small.* This dataset consists of 47 cases of soybean disease each characterized by 35 multivalued categorical variables. These cases are drawn from four populations, each one of them representing one of the following soybean diseases: D1-Diaporthe stem canker, D2-Charcoat rot, D3-Rhizoctonia root rot, and D4-Phytophthorat rot. We keep only 21 nonsingleton attributes.

*Mushroom Data.* Mushroom dataset consists of 8,124 data objects described by 22 categorical attributes distributed over 2 classes. The two classes are edible (4208 objects) and poisonous (3916 objects). It has missing values in attribute 11.

*Zoo Data.* It has 101 instances described by 16 attributes and distributed into 7 categories. The first attribute contains a unique animal name for each instance and is removed because it is noninformative. All other characteristics attributes are Boolean except for the character attribute which corresponds to the number of legs that lies in the set {0,2, 4,5, 6,8}.

*Lung-Cancer Data.* This dataset contains 32 instances described by 56 attributes distributed over 3 classes with missing values in attributes 5 and 39.

*Breast-Cancer Data.* This data has 699 instances with 9 attributes. Each data object is labeled as benign (458 or 65.5%) or malignant (241 or 34.5%). There are 9 instances in attributes 6 and 9 that contain missing attribute values.

*Dermatology Data.* This dataset contains six types of skin diseases for 366 patients that are evaluated using 34 clinical attributes, 33 of them are categorical and one is numerical. The categorical attribute values signify degrees in terms of whether the feature is present and contain largest possible amount or relative intermediate values. In our experiment, we discretize the numerical attribute (representing the age of the patient) into 10 categories.

*Congressional Vote Data.* This dataset includes votes for each of the US House of Representatives Congressmen on the 16 key votes. Each of the votes can either be a yes, no, or an unknown disposition. The data has 2 classes with 267 democrats and 168 republicans instances.

*Nursery.* This dataset was derived from a hierarchical decision model originally developed to rank applications for nursery schools. It contains 12,960 instances with 8 input attributes distributed over 5 classes. 

*Chess.* This dataset contains 3,196 instances, each of which is a board-description for the chess endgame with 36 features. Each game is labeled one of the two classes: “win” and “nowin.” 

*Heart Disease.* This dataset is the Cleveland heart disease database of 303 patients. The class represents presence of heart disease in the patient from 0 to 4. There are 13 attributes used in the experiments. We convert 5 numeric attributes (1st, 4th, 5th, 8th, and 10th) to categorical ones using the intervals of 10, 20, 60, 30, and 0.7, respectively. The 12th and 13th attributes contain missing attribute values.

Table 2 lists the characteristics of the chosen datasets. The columns avg.intra.dist and avg.inter.dist show the average intracluster and intercluster distances for each dataset, respectively.

(6) avg.intra.dist=∑k=1K∑x,y∈CkdHx,y∑k=1KCkCk−1/2avg.inter.dist=∑k=1K−1∑l=k+1K∑x∈Ck,y∈CldHx,y∑k=1K−1∑l=k+1KCkCl.

The column R shows the value of Hamming distance threshold estimated from the CDF (see Figure 3). AC is the accuracy of our CD-Clustering. We highlight the accuracy values that are larger than 0.8. The columns m, #comp, and top-*K* display the number of edges in *G*, the number of connected components in *G*, and the total number of data points in top-*K* clusters, respectively. Except Mushroom and Chess datasets, the top-*K* clusters detected by CD-Clustering include all or nearly all the data points. This result verifies the effectiveness of the simple estimation of *R* and the Louvain method. Finally, the column Runtime shows the runtime of CD-Clustering in millisecond which is almost linear in *m*.

### 5.2. Evaluation Metrics

To evaluate the performance of clustering algorithms, we use the same metrics as in [8, 9, 15]. If dataset contains *K* classes for a given clustering, let *a*_*i*_ denote the number of data objects that are correctly assigned to class *C*_*i*_, let *b*_*i*_ denote the data objects that are incorrectly assigned to the class *C*_*i*_, and let *c*_*i*_ denote the data objects that are incorrectly rejected from the class *C*_*i*_. The precision, recall, and accuracy are defined as

(7) PR=∑i=1Kai/ai+biK,RE=∑i=1Kai/ai+ciK,AC⁡=∑i=1KaiN.

We demonstrate how to find the best confusion matrix and compute the precision, recall, and accuracy metrics in the following example.

**Example 3 .**

Assume that a dataset of *N* = 10 objects clustered in *K* = 3 clusters with the ground-truth and predicted cluster labels as in Table 3.

To find the best confusion matrix *C*, we evaluate *K*! mappings from the set predicted labels {a, b, c} to the ground-truth {1,2, 3}. For example, the mapping {*c* → 1, *a* → 2, *b* → 3} gives us the confusion matrix in Table 4. The value in each cell, for example, (*c*, 1), is the number of pairs (*c*, 1) appearing in Table 3. Note that the sum of each column is equal to the number of objects in the corresponding cluster. The values of precision, recall, and accuracy are

(8) PR=1300+1+1+11+1+2+02+2+0=0.0833RE=1300+1+2+11+1+2+01+2+0=0.0833AC⁡=0+1+010=0.1000.

The best mapping in this case is {*b* → 1, *c* → 2, *a* → 3} with PR = 0.5000, RE = 0.5278, and AC⁡ = 0.5000.

### 5.3. Clustering Results

For comparison, we choose the algorithms by Cao et al. [8] and Khan and Ahmad [9] as well as the random *k*-modes [3]. We rerun the Java implementation provided in [9] and get a confusion matrix for each dataset. Then we find the best evaluation metrics using the brute-force technique in Example 3. Surprisingly, the metrics for seven datasets reported in [9] are not so good. They only match for the case of Vote data and get better value for Soybean and worse values on the other five datasets. For Cao's algorithm, our C++ implementation provides the matching results on three out of four datasets tested in [8], namely, Soybean, Mushroom, and Breast-Cancer. The worse metrics appear on Zoo data. However, our results agree with the Python implementation of Cao's algorithm at [27]. The results for random *k*-modes with 10,000 runs/dataset are also more or less different from [8, 9].

The clustering results for the ten categorical datasets are summarized in Tables 5–14. In terms of accuracy, precision, and recall, our scheme achieves the following results:

- Accuracy: our scheme outperforms or equals other methods in 7 cases, in particular with large margins in Lung-Cancer, Breast-Cancer, Dermatology, and Nursery datasets.
- Precision: our scheme outperforms or equals other methods in 7 cases.
- Recall: our scheme outperforms or equals other methods in 7 cases.

To better understand the performance of CD-Clustering, we revisit Table 2. There is a strong correlation between the accuracy metric AC⁡ and the gap between *R*, avg.intra.dist and avg.inter.dist. If the ground-truth average intracluster and intercluster distances are far apart and *R* is close to the former distance, we can get high accuracy (larger than 0.8). This is the cases of Soybean, Zoo, Breast-Cancer, Dermatology, and Vote. The two datasets with the lowest accuracy Nursery and Heart have smallest gaps between the ground-truth intracluster and intercluster distances. The three remaining datasets have medium accuracy despite the small distance gap. Out of the ten datasets, our CD-Clustering performs worst, that is, only comparable to or worse than the random *k*-modes, on Mushroom and Chess. This is reflected in the ratio of top-*K* to *N*: 5,366/8,124 (Mushroom) and 2,389/3,196 (Chess). Also, *K* is equal to 2 in these two datasets. These facts suggest that when *K* and the intra/intercluster distance gap are both small, CD-Clustering must struggle harder for the top-*K* communities.

## 6. Conclusion

Rather than using the *k*-modes algorithm with heuristic initialization methods, we propose in this paper a novel clustering scheme CD-Clustering for categorical data. By applying the Louvain method, a widely used community detection technique, CD-Clustering can uncover the highly homogeneous groups of categorical data points using only the distance information. CD-Clustering builds the simple graph by limiting all pairwise Hamming distances by a threshold *R* which is estimated simply using the number of clusters and the distance distribution. The evaluation against two *k*-modes initialization techniques confirms the effectiveness of CD-Clustering. In future work, we plan to reduce the complexity of CD-Clustering for better scalability.

## Figures and Tables

**Algorithm 1:**
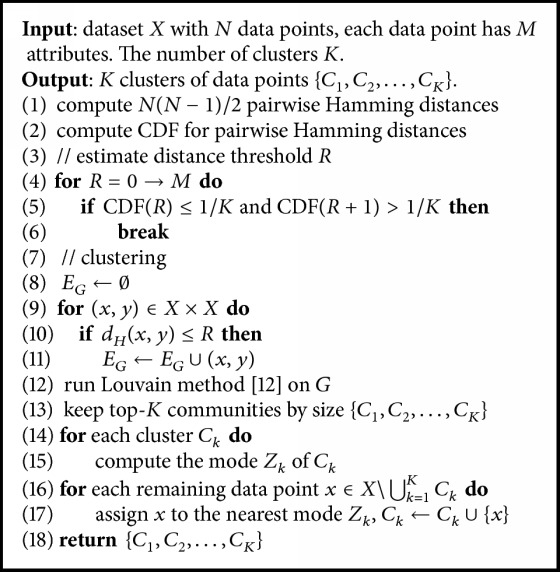
CD-Clustering.

**Table 1:** Comparison of time complexity.

Clustering method	Time complexity
Cao et al. [8]	*O*(*NMK*^2^)
Khan and Ahmad [9]	*O*(*NM* + *KM*^2^*N* + *N*log⁡*N*)
CD-Clustering	*O*(*N*^2^*M* + *N*^2^/*K*)

**Table 2:** Dataset properties.

Dataset	*N*	*M*	*K*	*R*	avg.intra.dist	avg.inter.dist	AC	*m*	#comp	top-*K*	Runtime (ms)
Soybean	47	21	4	7	5.54	12.48	**1.0000**	246	3	47	31
Mushroom	8,124	22	2	11	10.11	12.68	0.7244	12,924,407	1	5,366	8,284
Zoo	101	16	7	2	2.40	7.75	**0.8218**	701	7	100	109
Lung-Cancer	32	56	3	22	24.20	26.09	0.5938	160	4	29	110
Breast-Cancer	699	9	2	6	4.27	7.84	**0.9514**	115,068	1	699	93
Dermatology	366	34	6	10	11.23	16.41	**0.8552**	9,588	4	364	125
Vote	435	16	2	8	6.41	10.81	**0.8713**	47,102	1	432	93
Nursery	12,960	8	5	3	5.03	5.67	0.4156	5,721,840	1	12,960	2,543
Chess	3,196	36	2	9	9.65	10.35	0.6004	2,395,174	1	2,389	2,012
Heart	303	13	5	5	6.58	7.85	0.4719	7,755	2	302	94

**Table 3:** Ground-truth and predicted labels.

Object id	1	2	3	4	5	6	7	8	9	10
Ground-truth label	1	1	1	2	2	2	2	3	3	3
Predicted label	b	b	a	b	a	c	b	a	a	c

**Table 4:** A confusion matrix.

	1	2	3
c	**0**	1	1
a	1	**1**	2
b	2	2	**0**

**Table:** (a) Confusion matrix

	Class			
	D1	D2	D3	D4
D1	10	0	0	0
D2	0	10	0	0
D3	0	0	10	0
D4	0	0	0	17

**Table:** (b) Performance comparison

	Random	Cao	Khan	Proposed
AC	0.8044	**1**	0.9787	**1**
PR	0.7969	**1**	0.9773	**1**
RE	0.8005	**1**	0.9853	**1**

**Table:** (a) Confusion matrix

	Class	
	Poisonous	Edible
Poisonous	4093	2124
Edible	115	1792

**Table:** (b) Performance comparison

	Random	Cao	Khan	Proposed
AC	0.7206	**0.8754**	0.8288	0.7244
PR	0.7448	**0.9019**	0.8688	0.7990
RE	0.7167	**0.8709**	0.8228	0.7151

**Table:** (a) Confusion matrix

	Class						
	a	b	c	d	e	f	g
a	37	0	0	0	0	0	0
b	0	13	0	0	0	0	1
c	0	0	20	0	0	0	1
d	0	0	0	9	8	0	0
e	4	0	0	0	0	0	0
f	0	0	0	0	0	4	3
g	0	0	0	1	0	0	0

**Table:** (b) Performance comparison

	Random	Cao	Khan	Proposed
AC	0.7041	0.6733	**0.8614**	0.8218
PR	0.5876	0.5996	**0.7390**	0.5688
RE	0.5893	0.6233	**0.7648**	0.6861

**Table:** (a) Confusion matrix

	Class		
	a	b	c
a	5	2	2
b	5	7	1
c	3	0	7

**Table:** (b) Performance comparison

	Random	Cao	Khan	Proposed
AC	0.5227	0.5313	0.4375	**0.5938**
PR	0.5590	0.5833	0.4468	**0.5980**
RE	0.5283	0.5393	0.4470	**0.6208**

**Table:** (a) Confusion matrix

	Class	
	Benign	Malignant
Benign	432	8
Malignant	26	233

**Table:** (b) Performance comparison

	Random	Cao	Khan	Proposed
AC	0.8174	0.9113	0.6323	**0.9514**
PR	0.8283	0.9292	0.5535	**0.9407**
RE	0.7996	0.8773	0.5336	**0.9550**

**Table:** (a) Confusion matrix

	Class					
	Seborrheic dermatitis	Psoriasis	Lichen planus	Chronic dermatitis	Pityriasis rosea	Pityriasis rubra pilaris
Seborrheic dermatitis	61	0	2	0	49	0
Psoriasis	0	111	0	0	0	0
Lichen planus	0	0	70	0	0	0
Chronic dermatitis	0	1	0	52	0	0
Pityriasis rosea	0	0	0	0	0	1
Pityriasis rubra pilaris	0	0	0	0	0	19

**Table:** (b) Performance comparison

	Random	Cao	Khan	Proposed
AC	0.5683	0.5984	0.6175	**0.8552**
PR	0.5318	0.5548	0.6841	**0.7543**
RE	0.5028	0.5393	0.6165	**0.8189**

**Table:** (a) Confusion matrix

	Class				
	Not_recom	Recommend	Very_recom	Priority	Spec_prior
Not_recom	1440	0	132	1484	1264
recommend	0	0	0	0	0
Very_recom	0	0	0	0	0
Priority	1440	2	196	1924	758
Spec_prior	1440	0	0	858	2022

**Table:** (b) Performance comparison

	Random	Cao	Khan	Proposed
AC	0.3331	0.3673	0.2804	**0.4156**
PR	0.2902	0.2978	0.2304	**0.6494**
RE	**0.2592**	0.2273	0.2044	0.2569

**Table:** (a) Confusion matrix

	Class		
	Republican		Democrat
Republican	160		48
Democrat	8		219

**Table:** (b) Performance comparison

	Random	Cao	Khan	Proposed
AC	0.8603	0.8644	0.8506	**0.8713**
PR	0.8554	0.8568	0.8484	**0.8670**
RE	0.8732	0.8730	0.8672	**0.8863**

**Table:** (a) Confusion matrix

	Class		
	Win		Nowin
Win	1562		0
Nowin	1277		357

**Table:** (b) Performance comparison

	Random	Cao	Khan	Proposed
AC	0.6390	**0.7400**	0.7040	0.6004
PR	0.5184	0.5449	0.5312	**0.6092**
RE	0.5394	0.5806	0.5540	**0.7751**

**Table:** (a) Confusion matrix

	Class				
	0	1	2	3	4
0	99	9	0	1	0
1	24	10	4	2	2
2	14	25	30	28	8
3	20	9	1	2	1
4	7	2	1	2	2

**Table:** (b) Performance comparison

	Random	Cao	Khan	Proposed
AC	0.3895	0.3069	0.4422	**0.4719**
PR	0.3159	0.2763	**0.3413**	0.3271
RE	0.3219	0.2641	0.3467	**0.3660**

## Notations

*X* = {*X*_1_, *X*_2_,…, *X*_*N*_}: Dataset with *N* data points
*A* = {*A*_1_, *A*_2_,…, *A*_*M*_}: Set of *M* categorical attributes
{*Z*_1_,…, *Z*_*K*_}: *K* modes of *X*
*K*: Number of clusters
*d*_*H*_(*x*, *y*): Hamming distance between *x* and *y*
*R*: Hamming distance threshold
*G*(*X*, *R*): Simple graph for *X* with parameter *R*.
